# Embryo transfer in competition horses: Managing mares and expectations

**DOI:** 10.1111/eve.12182

**Published:** 2014-04-03

**Authors:** M L H Campbell

**Affiliations:** Department of Production and Population Health, The Royal Veterinary CollegeNorth Mymms, Hertfordshire, UK

**Keywords:** horse, equine embryo transfer, equine embryo recovery rates, stress, heat, exercise, transport

## Abstract

Embryo transfer (ET) is an accepted and successful technique for obtaining foals from mares without interrupting their competition careers. Recent research, however, suggests that the potential of factors including heat, exercise, repeated embryo flushing and repeated manipulation of the reproductive cycle using exogenous hormones to have a negative impact on fertility may have been underestimated. This paper reviews the evidence base for involvement of these factors in repeated failures to recover embryos from nongeriatric competition mares without obvious clinical or pathological indications of reproductive abnormalities. It concludes that, for some mares at least, a cessation of exercise for the periovulatory period and the period between ovulation and embryo flushing, combined with careful management of flushing-induced endometritis, and minimal hormonal manipulation of the reproductive cycle, may be necessary to optimise embryo recovery rates. Mare owners may have been encouraged to request ET for their mares following high-profile examples in the media of elite mares that have produced foals by ET whilst competing. The veterinarian should educate mare owners about the multiple factors that may affect the chances of recovering an embryo from their mares, and should manage the expectations of mare owners so that they do not approach ET programmes in the expectation that there will be no disruption to their training and competition plans.

## Introduction

The outgoing Chair of the UK's Human Embryology and Fertility Authority, Professor Lisa Jardine, recently commented that the assisted reproductive industry is ‘a market in hope’. ‘I would have loved’ she said on BBC's Point of View programme (BBC Radio 4 Point of View, 27 October 2013), ‘to have been able to have spoken more often and more publicly with words of caution’.

Equine embryo transfer (ET) has long been promoted as a means of breeding from competition mares before they undergo an age-associated reduction in fertility, without interrupting their athletic careers (Squires *et al.* 1999; Sitzenstock *et al.* 2013). This has been particularly beneficial in mares competing in sports such as dressage and eventing, in which many years of training are necessary before horses reach elite levels of competition. High-profile examples of competition mares producing foals by ET have helped to increase the uptake of ET technology amongst mare owners, and to persuade them that they can have the best of both worlds by reaping the simultaneous benefits of their mares' competitive and reproductive success.

Industry-wide, ET has undoubtedly proved a commercial success, and a useful tool for breeding from competition mares. What was, in the early days, a surgical technique with associated risks for the recipient mare has been refined, over the years, into a nonsurgical technique (Squires *et al.* 1982; Vanderwall and Woods 2007). There remains a persistent lack of a reliable, commercially available means of superovulating mares (Squires *et al.* 1999; Hinrichs 2012). Difficulties with freezing and thawing equine embryos (Stout 2012) have meant that in practice it is still commonly necessary to have a synchronised donor mare available at the time of embryo flushing, which is expensive. Despite these limitations, nonsurgical ET offers a good chance of producing a recipient pregnancy and a live birth of the donor's foal (Hartman 2011).

Notwithstanding this overall success, however, many of us who work in the field of equine ET can readily recall cases in which we have struggled to recover embryos from mares that, on paper, ought to have been ideal embryo donors. Given the economic investment that owners spend on artificial insemination and ET programmes, the planning required to fit around competition schedules, and the time veterinarians spend organising and executing the reproductive procedures, such cases can prove very frustrating for everyone concerned.

Embryo transfer is probably the most widely used artificial reproductive technique in mares other than artificial insemination (Hartman 2011). Yet its widespread practice is increasingly tempered by research into management factors affecting fertility (Mortensen *et al.* 2009; Vazquez *et al.* 2010; Kelley *et al.* 2011; Smith *et al.* 2012; Fazio *et al.* 2013), which suggests that breeding from competition mares may not be as straightforward as early advocates of ET suggested. The combined implication of a number of recent papers reviewed below is to suggest that Professor Jardine's ‘words of caution’ might be justified when managing competition mare owners' expectations. Disruption to training and competition schedules, for some mares at least, may be necessary to optimise embryo recovery rates. The aims of this paper are to review the evidence base about management factors that might limit embryo recovery rates in nongeriatric competition mares, and to suggest how mare management might be adapted to minimise the impact of those factors.

## Success rates with equine nonsurgical ET

According to a survey presented by Patrick McCue of Colorado State University for the 2010 American Association of Equine Practitioners Convention (McCue 2010), embryo recovery rates are affected by age and fertility of the donor mare, the quality of the sire's semen, the day of recovery, the number of ovulations, and clinical expertise. Embryo recovery rate is correlated with age and reproductive status of the donor mare. A higher percentage of embryos are recovered from mares aged <10 years than from mares aged >15 years (McCue 2010). Any reproductive abnormality of the donor mare inevitably reduces embryo recovery rates. Endometritis of the donor mare, although it does not necessarily preclude embryo recovery, reduces the chances of the embryo surviving in the recipient mare after transfer (Hartman 2011).

Generally, assuming clinical competence, if there is an embryo there then it will be recovered (McCue 2010; Hartman 2011). Recovery rates are therefore broadly reflective of per cycle conception rates that, industry-wide and dependent upon clinical competence and mare and stallion variation, are around 60–77% for fresh, 44% for chilled and 46% for frozen semen (Squires *et al.* 2006).

## Definition of ‘competition’ mares

The mares of interest for the purposes of this review are those from which there is a repeated failure to recover an embryo (or much lower recovery rates than expected) despite them being nongeriatric and in good general health; medication-free (other than reproductive hormones – see below); having no identified systemic illness or lameness issues; having no detectable abnormalities on a breeding soundness examination, and being inseminated and flushed by a competent veterinarian using good quality semen of known fertility. Often, these are mares participating in disciplines such as 3-day eventing, reining, endurance, polo, dressage and showjumping. Mares in which there is a detectable reproductive abnormality or management issue that explains failure to recover an embryo at the time of flushing are not considered in this paper.

## Possible explanations for unexpectedly low embryo recovery rates in competition mares

It seems increasingly possible that the ‘fertility of the donor mare’ identified as one of the factors limiting embryo recovery rates (McCue 2010) is affected by the way in which we manage competition mares in ET programmes.

### Repeated embryo recovery attempts

Repeated acute endometritis may result in chronic degenerative fibrotic changes to the equine endometrium (Hoffman *et al.* 2009). One study (Aurich *et al.* 2011) demonstrated no alteration in the embryo recovery rate with an increasing number of successive embryo collections. However, other work has demonstrated a positive correlation between repeated embryo recovery attempts and chronic inflammatory changes in the uterus (Carnevale *et al.* 2005). In clinical practice there is ‘substantial evidence’ that repeated embryo recovery attempts can result in acute bacterial endometritis and chronic endometrial inflammation (Hartman 2011). Repeated embryo recovery attempts may therefore result in a reduction in embryo recovery rates from a particular mare over time, and flushing-induced endometritis can limit the number of embryos recovered from a mare in one year. Some authors also contend that the fact that mares used as donors for many years never foal themselves has a detrimental effect on the ability of their cervices to dilate, making them increasingly prone to endometritis (Riera 2011).

### The effect of heat on fertility

The effect of exercise, which caused an increase in rectal temperature (from 38°C to a mean of 39.9°C), on mare reproductive efficiency was evaluated by comparing rates of embryo recovery from mares assigned to either an exercise regimen or a nonexercise (control) regimen (Mortensen *et al.* 2009). Exercised mares were worked daily for 30 min under average ambient conditions of >30°C and >50% humidity. Mares were inseminated during oestrus and subjected to uterine flush for embryo recovery on Day 7 after ovulation for 2 consecutive cycles. After this, mares were allocated to the opposite group and allowed an oestrous cycle without reproductive manipulation. Insemination and uterine flushing were then conducted on 2 more consecutive cycles. Embryo recovery from control mares was 63%. Overall, there was an almost 50% reduction in embryo recovery rates in mares that were exercised throughout their reproductive cycle under hot and humid conditions. Fewer embryos were recovered from exercised mares (34%) compared to control mares (63%) (P<0.05). The proportion of embryos classified as *grade 1* tended to be less in exercised than in nonexercised mares (36% compared to 73%; P = 0.051). These results are consistent with results for cattle, where heat stress during the periovulatory and fertilisation period has been shown to retard embryonic development and lead to lower quality embryos being recovered (Putney *et al.* 1988). The work of Mortensen *et al*. (2009) suggests that heat stress (due to exercise or other causes) can be a limiting factor in embryo recovery in competition mares.

### The effect of exercise on fertility

The effect of exercise as distinct from that of temperature on fertility (Smith *et al.* 2012) was assessed by exercising mares moderately in conditions that gave rise to a smaller increase in rectal temperature than that reported in the study of Mortensen *et al*. (2009). The study of Smith *et al*. (2012) also tried to determine whether, if exercise does have a detrimental effect on fertility, that negative effect is on early embryonic development or on conception. The study assessed the effect of differing exercise protocols on reproductive blood flow and embryo recovery and quality. Light-horse mares were randomised into control, partial-exercised and full-exercised groups. Partial-exercised mares were moderately exercised for 30 min daily during the periovulatory period, but rested after ovulation for 7 days. Full-exercised mares were exercised for 30 min every day of their reproductive cycle. All mares were artificially inseminated during oestrus, and embryo recovery was performed 7 days post ovulation. Blood flow through both ovarian arteries and vascular perfusion of the wall of the preovulatory follicle were examined by colour Doppler ultrasonography (Gastal 2011). Serum cortisol concentrations were measured in blood samples taken from all mares immediately before exercise and 30 min after completion of exercise for the first and final reproductive cycles.

The results of Smith *et al*. (2012) showed that, consistent with an earlier study (Kelley *et al.* 2011), exercise induced greater serum cortisol concentrations. Embryo recovery rates were 43% in exercised mares compared with 67% in control mares (P<0.10). However, embryo recovery rates for partial-exercised (44%) and full-exercised (43%) mares were not significantly different. Fewer *grade 1* embryos were recovered from partial-exercised mares compared with both control and full-exercised mares (P<0.05).

Blood flow through both ovarian arteries was greater in both exercised groups in the days leading up to ovulation (P<0.05) (Smith *et al.* 2012). However, vascular perfusion of the wall of the preovulatory follicle on the day before ovulation was less in both partial-exercised and fully exercised mares compared to control mares (P<0.05). In exercised mares, vascular perfusion of the follicle wall was positively correlated with the likelihood of recovering an embryo.

The findings of Smith *et al*. (2012) support previous work that has demonstrated a positive correlation between vascularity of the preovulatory follicle and pregnancy rates in cattle (Siddiqui *et al.* 2009) and horses (Silva *et al.* 2006; Gastal and Gastal 2011), and between vascularity of the preovulatory follicle and embryo recovery rate in women (Coulam *et al.* 1999).

Smith *et al*. (2012) concluded that ‘exercise…was detrimental to embryo recovery rate’. The results of the study by Smith *et al*. (2012) suggest that even quite moderate exercise under nonextreme conditions can reduce embryo recovery rates in mares. Furthermore, the effect of exercise on embryo recovery rates appears to be related to exercise during the periovulatory period. Because the negative effect of exercise on embryo recovery rates occurred whether or not mares were rested in the period between insemination and embryo flushing, it probably occurs at the level of conception, not of early embryo development. One possible explanation for exercise having a negative impact on conception is that exercise causes a general disruption of follicular development and maturation. A combination of mechanisms seems to be responsible for this disruption, which is reflected in decreased vascular perfusion of the wall of the preovulatory follicle (Gastal and Gastal 2011; Smith *et al.* 2012). Exercise increases circulating cortisol levels (Kelley *et al.* 2011; Smith *et al.* 2012) and reduces plasma concentrations of luteinising hormone (LH) (Kelley *et al.* 2011). Increased follicle blood flow, along with a rapid increase in LH at the terminal stage of follicle maturation, is associated with successful meiosis resumption and normal completion of oocyte maturation (Gastal and Gastal 2011). Hence exercise-induced reduction in LH levels and vascular perfusion of the preovulatory follicle wall result in a reduced ability of the largest follicle to exert its dominance, delayed ovulation and increased interovulatory intervals (Kelley *et al.* 2011).

In contrast to the studies described above, work by Vazquez *et al*. (2010) and Pessoa *et al*. (2011) found that exercise under hot and humid conditions had no effect on embryo recovery rates. This discrepancy in the literature is perhaps unsurprising since anecdotally embryo recovery rates are good in some mares who continue to train and compete hard throughout an ET programme, suggesting that not all mares suffer a reduction in fertility as a result of heat or exercise stress. However, in some mares at least exercise does seem to have a disruptive effect on follicular development and dynamics. In those mares where this does occur, it is likely not only to reduce pregnancy/embryo recovery rates (Silva *et al.* 2006; Gastal and Gastal 2011) but also (particularly where the number of recipients available is limited) to complicate management of ET competition mares by making synchronisation of donors and recipients more difficult.

Pessoa *et al*. (2011) suggest that one possible explanation for the fact that they failed to demonstrate an effect of exercise on embryo recovery rates whereas other authors demonstrated a negative effect (Smith *et al.* 2012) was that the mares in the study of Pessoa *et al*. (2011) were fitter, and therefore experienced a smaller increase in rectal temperature as the result of exercise. The suggestion that being fit increases embryo recovery rates in mares is an interesting one. In women, to the contrary, even though exercise has been shown to have beneficial effects on reproductive health, excessive exercise can cause ovarian dysfunction (for review, see Orio *et al.* 2013). Perhaps the implication of the findings of Pessoa *et al*. (2011) is not so much that extreme fitness improves conception rates in mares as that lack of fitness combined with exposure to exercise can reduce them, by increasing the likelihood of exercise-associated heat stress.

The fact that experimentally interovulatory intervals are increased by exercise (Kelley *et al.* 2011) might support the idea that fitness in mares has some bearing on ovarian function. One way of minimising the effect of this possibility would be to schedule ET attempts for outside the mare's busy training and competition season. That would also minimise any negative effects on embryo recovery rate of exercise in the insemination/flushing period. Unfortunately, for the majority of equine sports, the competitive off-season is the winter, when most mares (being seasonal breeders) are not cycling. The effect of season on ET in mares is somewhat unclear. Aurich *et al*. (2011) reported no difference in embryo recovery rates at different times of year. However, pregnancy rates once embryos have been transferred into recipients may be affected by season: Squires *et al*. (1982) reported that their success rates were consistently lower across years for embryos transferred between March and June than for July to October. Those authors suggested that recipient mares might need to undergo one or 2 normal oestrus cycles within the physiological breeding season before pregnancy rates post transfer could be optimised.

Later work that reported the successful use of ovariectomised mares (Hinrichs *et al.* 1985) or seasonally anoestrus mares treated with exogenous hormones (Kaercher *et al.* 2013) as ET recipients somewhat calls into question this suggestion of Squires *et al*. (1982). Nonetheless, the possibility remains that ET overall may be more successful during the physiological breeding season than outside it. At the very least, given the general difficulty in getting mares to cycle reliably outside of the physiological breeding season (see, for example, Nagy *et al.* 2000 and Murphy *et al.* 2014), synchronisation of donor and recipient may be easier during the physiological breeding season than outside it. This managerial factor is particularly important when there are only a limited number of recipients available per donor.

### The effect of transportation on embryo recovery rates

The effects of exercise on circulating cortisol levels in the studies described above, and the consequent negative effect on fertility, suggest that anything that causes an increase in cortisol has the potential to reduce equine pregnancy/embryo recovery rates. The hypothalamic-pituitary-adrenal axis is activated by stress, physiological or psychological. In women, psychological stress is correlated with ovarian dysfunction, caused by high circulating stress hormones interfering with the timing of ovulation and shortening the luteal phase (Nakamura 2008). Activation of the hypothalamic-pituitary-adrenal axis exerts an inhibitory effect on the female reproductive system. This is because corticotropin-releasing hormone inhibits hypothalamic gonadotropin-releasing hormone secretion, and glucocorticoids also inhibit pituitary luteinising hormone, ovarian oestrogen and ovarian progesterone secretion. This mechanism is essentially the same as that implicated in disrupting ovarian function in exercised mares described above (Kelley *et al.* 2011). This raises the possibility that psychological as well as physical stress might have a negative effect on embryo recovery rates from competing mares. Although earlier studies failed to demonstrate an effect of transportation on pregnancy rates (Baucus *et al.* 1990; Berghold *et al.* 2007), it is well recognised that transportation causes an increase in plasma cortisol levels in horses (Fazio *et al.* 2008), even when journey distances are short (Fazio *et al.* 2013). Tischner *et al*. (2006) measured stress responses to transportation in mares at different stages of the oestrus cycle and of pregnancy. These authors reported that ‘the most intensive stress reaction (to transportation), measured by maximum rise in noradrenaline, adrenaline and cortisol, was shown in mares in diestrus and during the winter anestrus’. This suggests that competition mares being subjected to embryo transfer procedures could be particularly susceptible to stress if transported in the interval between insemination and flushing, when they are in dioestrus.

Given the negative effect of heat on equine embryo recovery rates demonstrated by Mortensen *et al*. (2009)*,* it seems possible that the combined effect of stress and heat in mares that are ‘bad travellers’ is another factor that could limit embryo recovery rates in competition mares.

### The effect of repeated manipulation of the reproductive cycle on embryo recovery rates

Equine ET frequently involves manipulation of the reproductive cycle using exogenous hormones. This occurs particularly when the number of recipient mares per donor is limited (as is not uncommon in the UK), and/or when the intention is to maximise the number of embryos that can be recovered from a donor in one breeding season. Prostaglandin F2α is commonly used both to synchronise donors and recipients, and to short-cycle donors to re-breed them as quickly as possible following an embryo flush. One study suggested that use of PGF2α to induce oestrus is associated with reduced pregnancy rates in mares (Nielsen *et al.* 2008). However, other authors found no difference in pregnancy rates between mares in which oestrus had been induced using PGF2α and control mares (Veronesi *et al.* 2003; Metcalf and Thompson 2010). It is therefore unclear whether the repeated use of PGF2α might be a contributing factor to unexplained low embryo recovery rates in some competition mares, particularly because different analogues of PGF2α were used in different studies, and the response of mares to PGF2α anyway varies depending upon the stage of their reproductive cycle at which it is administered (Newcombe *et al.* 2008). Given that induction of oestrus is common in most equine reproductive practices, and that rates of embryo recovery are generally good, it seems unlikely that use of PGF2α generally exerts a significant negative effect on embryo recovery rates. However, the possibility remains that some mares to which PGF2α is repeatedly administered may suffer reduced pregnancy rates, and it is also true that if mares ovulate very soon after the administration of PGF2α (Newcombe *et al.* 2008) they may do so without the uterus undergoing much exposure to the low-progesterone-high-oestrogen environment usually associated with oestrus. This potentially has a negative effect on early embryo development (although this effect may be more important in recipients that have been short-cycled using PGF2α than it is in donors).

## Implications for mare management

There is a dearth of published large-scale, clinical research studies from commercial ET centres looking at the effects of exercise, heat and stress in competition mares. Data from such studies comparing embryo recovery rates for the same competition mare undergoing embryo flushing whilst at competition fitness and during periods of rest would be useful additions to the evidence base on this subject. Nonetheless, the research papers reviewed above combine to suggest that mare management can affect embryo recovery rates. It follows that, ideally, mares would not be exercised, nor exposed to events likely to induce heat-stress, psychological stress or physical stress, either during the periovulatory period (which seems to be most crucial), or during the time between insemination and embryo flushing. Equally, mares that are bad travellers would not be transported (for example to and from a clinic) in the periovulatory period, or during dioestrus post insemination. Embryo transfer procedures would probably ideally be scheduled during the physiological breeding season. In those mares in which repeated embryo recovery attempts induce endometritis, uterine inflammation would be controlled between ET cycles to optimise embryo recovery and recipient pregnancy rates. Furthermore, since a negative effect of repeated manipulation of the reproductive cycle of donors and recipients on embryo recovery rates and survival rates post transfer cannot be ruled out, hormonal manipulation of the reproductive cycle would be minimised (which means that the number of potential recipients per donor would be high).

## Conclusions

Embryo transfer *is* a useful and efficient means of breeding from mares that are still training and competing. The science on the effects of exercise and heat on conception is equivocal, and good embryo recovery rates industry-wide suggest that in many cases mares manage to conceive repeatedly and to donate embryos despite the stresses of training, competition and transportation. This is fortunate since the ideal management conditions specified above would be difficult to meet in the situation where large herds of recipient mares are few and far between, and donor mares need to keep competing.

Nonetheless, mare owners should not approach ET programmes in the expectation that there will be no disruption to their training and competition plans. It is in the interests of the owners as well as the veterinarians involved for everyone to understand at the outset that, to echo Professor Jardine's sentiments, ET has the potential to deliver disappointment as well as success. The research reviewed in this paper has elucidated the potential for exercise, heat and stress to disrupt ovarian function. In mares from which there is a repeated failure of embryo recovery in the absence of clinically detectable reproductive abnormality, these factors should be considered. For some mares at least, owners may have to accept that alterations to management are necessary, and that training as well as competition may have to cease entirely during the periovulatory period and the gap between insemination and flushing. Furthermore, to minimise the possible negative effect of repeated hormonal manipulations of the reproductive cycle on pregnancy rates, even those owners who are prepared to give their mares an ‘easy week’ around the time of insemination and flushing may need to be flexible about when that week occurs, rather than to expect to be able to dictate it based solely around competition schedules.

## Author's declaration on interests

The author is funded by the Wellcome Trust as Clinical Research Fellow in Veterinary Ethics at the Royal Veterinary College.
